# Supplementary material for: Resolving the full spectrum of human genome variation using Linked-Reads
Source: Genome Res. 2019 Apr;29(4):635–45. doi: 10.1101/gr.234443.118 (PMC6442396; doi:10.1101/gr.234443.118)
Supplement: Supplemental Material [file supp_gr.234443.118_Supplemental_longranger-2.2.zip › longranger-2.2.tar.bz2!/lr2.2-public/lib/stan/cmdstan/stan/doc/api/html/functions_a.html]

Stan: Class Members


|  |  |
| --- | --- |
|  | Stan  2.5.0  probability, sampling & optimization |


- Main Page
- Related Pages
- Namespaces
- Classes
- Files

- Class List
- Class Index
- Class Hierarchy
- Class Members

- All
- Functions
- Variables
- Typedefs
- Enumerator
- Related Functions

- \_
- a
- b
- c
- d
- e
- f
- g
- h
- i
- j
- k
- l
- m
- n
- o
- p
- q
- r
- s
- t
- u
- v
- w
- x
- y
- z
- ~


All Classes Namespaces Files Functions Variables Typedefs Enumerations Enumerator Friends Macros Pages

Here is a list of all class members with links to the classes they belong to:

### - a -

- accept\_stat()
  : stan::mcmc::sample
- accumulator()
  : stan::math::accumulator< T >
- ad\_
  : stan::agrad::op\_ddv\_vari
  , stan::agrad::op\_dv\_vari
  , stan::agrad::op\_dvd\_vari
  , stan::agrad::op\_dvv\_vari
- adapt\_base\_window\_
  : stan::mcmc::windowed\_adaptation
- adapt\_dense\_e\_nuts()
  : stan::mcmc::adapt\_dense\_e\_nuts< M, BaseRNG >
- adapt\_dense\_e\_static\_hmc()
  : stan::mcmc::adapt\_dense\_e\_static\_hmc< M, BaseRNG >
- adapt\_diag\_e\_nuts()
  : stan::mcmc::adapt\_diag\_e\_nuts< M, BaseRNG >
- adapt\_diag\_e\_static\_hmc()
  : stan::mcmc::adapt\_diag\_e\_static\_hmc< M, BaseRNG >
- adapt\_flag\_
  : stan::mcmc::base\_adapter
- adapt\_init\_buffer\_
  : stan::mcmc::windowed\_adaptation
- adapt\_next\_window\_
  : stan::mcmc::windowed\_adaptation
- adapt\_term\_buffer\_
  : stan::mcmc::windowed\_adaptation
- adapt\_unit\_e\_nuts()
  : stan::mcmc::adapt\_unit\_e\_nuts< M, BaseRNG >
- adapt\_unit\_e\_static\_hmc()
  : stan::mcmc::adapt\_unit\_e\_static\_hmc< M, BaseRNG >
- adapt\_window\_counter\_
  : stan::mcmc::windowed\_adaptation
- adapt\_window\_size\_
  : stan::mcmc::windowed\_adaptation
- adaptation
  : stan::io::stan\_csv
- adaptation\_window()
  : stan::mcmc::windowed\_adaptation
- adapting()
  : stan::mcmc::base\_adapter
- add()
  : stan::gm::function\_signatures
  , stan::gm::variable\_map
  , stan::math::accumulator< T >
  , stan::math::array\_builder< T >
  , stan::mcmc::chains< RNG >
- add\_binary()
  : stan::gm::function\_signatures
- add\_nullary()
  : stan::gm::function\_signatures
- add\_quaternary()
  : stan::gm::function\_signatures
- add\_sample()
  : stan::prob::welford\_covar\_estimator
  , stan::prob::welford\_var\_estimator
- add\_ternary()
  : stan::gm::function\_signatures
- add\_unary()
  : stan::gm::function\_signatures
- add\_vec()
  : stan::io::var\_context
- AddCost
  : Eigen::NumTraits< stan::agrad::var >
- adj()
  : stan::agrad::var
- adj\_
  : stan::agrad::vari
- algorithm
  : stan::io::stan\_csv\_metadata
- all\_constant
  : stan::agrad::OperandsAndPartials< T1, T2, T3, T4, T5, T6, T\_return\_type >
- all\_partials
  : stan::agrad::OperandsAndPartials< T1, T2, T3, T4, T5, T6, T\_return\_type >
- all\_varis
  : stan::agrad::OperandsAndPartials< T1, T2, T3, T4, T5, T6, T\_return\_type >
- alloc()
  : stan::memory::stack\_alloc
- alpha()
  : stan::optimization::BFGSMinimizer< FunctorType, QNUpdateType, Scalar, DimAtCompile >
- alpha0()
  : stan::optimization::BFGSMinimizer< FunctorType, QNUpdateType, Scalar, DimAtCompile >
  , stan::optimization::LSOptions< Scalar >
- append\_samples
  : stan::io::stan\_csv\_metadata
- apply()
  : stan::math::promote\_scalar\_struct< T, S >
  , stan::math::promote\_scalar\_struct< T, Eigen::Matrix< S, 1,-1 > >
  , stan::math::promote\_scalar\_struct< T, Eigen::Matrix< S,-1, 1 > >
  , stan::math::promote\_scalar\_struct< T, Eigen::Matrix< S,-1,-1 > >
  , stan::math::promote\_scalar\_struct< T, std::vector< S > >
  , stan::math::promote\_scalar\_struct< T, T >
- arg()
  : stan::gm::argument
  , stan::gm::argument\_parser
  , stan::gm::categorical\_argument
  , stan::gm::list\_argument
- arg\_adapt()
  : stan::gm::arg\_adapt
- arg\_adapt\_delta()
  : stan::gm::arg\_adapt\_delta
- arg\_adapt\_engaged()
  : stan::gm::arg\_adapt\_engaged
- arg\_adapt\_gamma()
  : stan::gm::arg\_adapt\_gamma
- arg\_adapt\_init\_buffer()
  : stan::gm::arg\_adapt\_init\_buffer
- arg\_adapt\_kappa()
  : stan::gm::arg\_adapt\_kappa
- arg\_adapt\_t0()
  : stan::gm::arg\_adapt\_t0
- arg\_adapt\_term\_buffer()
  : stan::gm::arg\_adapt\_term\_buffer
- arg\_adapt\_window()
  : stan::gm::arg\_adapt\_window
- arg\_bfgs()
  : stan::gm::arg\_bfgs
- arg\_data()
  : stan::gm::arg\_data
- arg\_data\_file()
  : stan::gm::arg\_data\_file
- arg\_decl()
  : stan::gm::arg\_decl
- arg\_decl\_r
  : stan::gm::functions\_grammar< Iterator >
- arg\_decls\_
  : stan::gm::function\_decl\_def
- arg\_decls\_r
  : stan::gm::functions\_grammar< Iterator >
- arg\_dense\_e()
  : stan::gm::arg\_dense\_e
- arg\_diag\_e()
  : stan::gm::arg\_diag\_e
- arg\_diagnose()
  : stan::gm::arg\_diagnose
- arg\_diagnostic\_file()
  : stan::gm::arg\_diagnostic\_file
- arg\_engine()
  : stan::gm::arg\_engine
- arg\_fail()
  : stan::gm::arg\_fail
- arg\_fixed\_param()
  : stan::gm::arg\_fixed\_param
- arg\_history\_size()
  : stan::gm::arg\_history\_size
- arg\_hmc()
  : stan::gm::arg\_hmc
- arg\_id()
  : stan::gm::arg\_id
- arg\_init()
  : stan::gm::arg\_init
- arg\_init\_alpha()
  : stan::gm::arg\_init\_alpha
- arg\_int\_time()
  : stan::gm::arg\_int\_time
- arg\_iter()
  : stan::gm::arg\_iter
- arg\_lbfgs()
  : stan::gm::arg\_lbfgs
- arg\_max\_depth()
  : stan::gm::arg\_max\_depth
- arg\_method()
  : stan::gm::arg\_method
- arg\_metric()
  : stan::gm::arg\_metric
- arg\_newton()
  : stan::gm::arg\_newton
- arg\_num\_samples()
  : stan::gm::arg\_num\_samples
- arg\_num\_warmup()
  : stan::gm::arg\_num\_warmup
- arg\_nuts()
  : stan::gm::arg\_nuts
- arg\_optimize()
  : stan::gm::arg\_optimize
- arg\_optimize\_algo()
  : stan::gm::arg\_optimize\_algo
- arg\_output()
  : stan::gm::arg\_output
- arg\_output\_file()
  : stan::gm::arg\_output\_file
- arg\_random()
  : stan::gm::arg\_random
- arg\_refresh()
  : stan::gm::arg\_refresh
- arg\_rwm()
  : stan::gm::arg\_rwm
- arg\_sample()
  : stan::gm::arg\_sample
- arg\_sample\_algo()
  : stan::gm::arg\_sample\_algo
- arg\_save\_iterations()
  : stan::gm::arg\_save\_iterations
- arg\_save\_warmup()
  : stan::gm::arg\_save\_warmup
- arg\_seed()
  : stan::gm::arg\_seed
- arg\_static()
  : stan::gm::arg\_static
- arg\_stepsize()
  : stan::gm::arg\_stepsize
- arg\_stepsize\_jitter()
  : stan::gm::arg\_stepsize\_jitter
- arg\_test()
  : stan::gm::arg\_test
- arg\_test\_grad\_eps()
  : stan::gm::arg\_test\_grad\_eps
- arg\_test\_grad\_err()
  : stan::gm::arg\_test\_grad\_err
- arg\_test\_gradient()
  : stan::gm::arg\_test\_gradient
- arg\_thin()
  : stan::gm::arg\_thin
- arg\_tolerance()
  : stan::gm::arg\_tolerance
- arg\_type\_
  : stan::gm::arg\_decl
- arg\_unit\_e()
  : stan::gm::arg\_unit\_e
- args\_
  : stan::gm::array\_literal
  , stan::gm::distribution
  , stan::gm::fun
- args\_r
  : stan::gm::term\_grammar< Iterator >
- argument()
  : stan::gm::argument
- argument\_parser()
  : stan::gm::argument\_parser
- argument\_probe()
  : stan::gm::argument\_probe
- array()
  : stan::math::array\_builder< T >
- array\_builder()
  : stan::math::array\_builder< T >
- array\_dims\_r
  : stan::gm::bare\_type\_grammar< Iterator >
- array\_literal()
  : stan::gm::array\_literal
- array\_vec\_t
  : stan::io::writer< T >
- assignment()
  : stan::gm::assignment
- assignment\_r
  : stan::gm::statement\_grammar< Iterator >
- autocorrelation()
  : stan::mcmc::chains< RNG >
- autocovariance()
  : stan::mcmc::chains< RNG >
- available()
  : stan::io::reader< T >
- available\_i()
  : stan::io::reader< T >
- avi\_
  : stan::agrad::op\_v\_vari
  , stan::agrad::op\_vd\_vari
  , stan::agrad::op\_vdd\_vari
  , stan::agrad::op\_vdv\_vari
  , stan::agrad::op\_vv\_vari
  , stan::agrad::op\_vvd\_vari
  , stan::agrad::op\_vvv\_vari


---

[ Stan Home Page ]

*© 2011–2014,
Stan Development Team.*
